# Operational Characteristics of AlGaN/GaN High-Electron-Mobility Transistors with Various Dielectric Passivation Structures for High-Power and High-Frequency Operations: A Simulation Study

**DOI:** 10.3390/mi15091126

**Published:** 2024-09-03

**Authors:** Ji-Hun Kim, Chae-Yun Lim, Jae-Hun Lee, Jun-Hyeok Choi, Byoung-Gue Min, Dong Min Kang, Hyun-Seok Kim

**Affiliations:** 1 Division of Electronics and Electrical Engineering, Dongguk University-Seoul, Seoul 04620, Republic of Korea; kjsuk0105@dongguk.edu (J.-H.K.); 2021111862@dongguk.ac.kr (C.-Y.L.); leejae00@dongguk.edu (J.-H.L.); junhyeok6293@dgu.ac.kr (J.-H.C.); 2 Electronics and Telecommunications Research Institute, Daejeon 34129, Republic of Korea; minbg@etri.re.kr (B.-G.M.); kdm1597@etri.re.kr (D.M.K.)

**Keywords:** gallium nitride, high-electron-mobility transistor, passivation, dielectric material, breakdown voltage

## Abstract

This study investigates the operational characteristics of AlGaN/GaN high-electron-mobility transistors (HEMTs) by employing various passivation materials with different dielectric constants and passivation structures. To ensure the simulation reliability, the parameters were calibrated based on the measured data from the fabricated basic Si_3_N_4_ passivation structure of the HEMT. The Si_3_N_4_ passivation material was replaced with high-k materials, such as Al_2_O_3_ and HfO_2_, to improve the breakdown voltage. The Al_2_O_3_ and HfO_2_ passivation structures achieved breakdown voltage improvements of 6.62% and 17.45%, respectively, compared to the basic Si_3_N_4_ passivation structure. However, the increased parasitic capacitances reduced the cut-off frequency. To mitigate this reduction, the operational characteristics of hybrid and partial passivation structures were analyzed. Compared with the HfO_2_ passivation structure, the HfO_2_ partial passivation structure exhibited a 7.6% reduction in breakdown voltage but a substantial 82.76% increase in cut-off frequency. In addition, the HfO_2_ partial passivation structure exhibited the highest Johnson’s figure of merit. Consequently, considering the trade-off relationship between breakdown voltage and frequency characteristics, the HfO_2_ partial passivation structure emerged as a promising candidate for high-power and high-frequency AlGaN/GaN HEMT applications.

## 1. Introduction

AlGaN/GaN high-electron-mobility transistors (HEMTs) are increasingly being adopted for high-power applications due to their superior material properties, such as a wide energy bandgap (3.4 eV) and a high critical electric field (3.39 MV/cm). GaN exhibits a higher electron saturation velocity and current density than conventional silicon and gallium arsenide [1,2,3]. AlGaN/GaN heterostructure HEMTs exhibit exceptional performance due to the formation of a two-dimensional electron gas (2-DEG) at the surface, which arises from spontaneous and piezoelectric polarization effects [4,5]. Consequently, these HEMTs are extensively employed in power electronics and devices that operate under high-power and high-frequency conditions. To optimize these outstanding characteristics, we developed various dielectric passivation structures that enhance the breakdown voltage (V_BD_) and cut-off frequency (f_T_) simultaneously. After conducting the simulation for each structure, Johnson’s figure of merit (JFOM), which can be expressed as V_BD_ × f_T_, was used to evaluate the operational characteristics [6,7].

High-k materials are commonly used in the passivation layer because of their advantages. Due to its higher dielectric constant than conventional materials such as SiO_2_ and Si_3_N_4_, a high-k material functions as a thicker dielectric layer without physically increasing its thickness. It effectively reduces leakage current under both off-state and on-state conditions [8,9,10]. In addition, high-k materials contribute to the electric field redistribution, which improves the breakdown voltage of HEMT devices [11,12]. However, the large dielectric constant of these materials also increases parasitic capacitances, such as the gate-to-source capacitance (C_GS_) and gate-to-drain capacitance (C_GD_), which can degrade the frequency characteristics [13]. Therefore, it is crucial to balance the trade-off between V_BD_ and frequency characteristics when selecting high-k materials for the passivation layer of AlGaN/GaN HEMTs [14,15].

In this study, we simulated and analyzed three distinct passivation structures: entire passivation (EP), hybrid passivation (HP), and partial passivation (PP) structures, using different dielectric materials such as Si_3_N_4_, Al_2_O_3_, and HfO_2_ to improve the V_BD_ with minimal degradation in frequency characteristics. The JFOM was calculated and analyzed for seven different structures in terms of the trade-off relationship between V_BD_ and f_T_. First, the Al_2_O_3_ and HfO_2_ EP structures were modeled by substituting the passivation material of the basic Si_3_N_4_ passivation structure. We confirmed that the EP structure with HfO_2_ passivation, which has the highest dielectric constant among the dielectric materials, exhibited the highest V_BD_ because it effectively redistributed the electric field when a high drain voltage (V_DS_) was applied. Conversely, when a high-k material was applied as the passivation layer, the parasitic capacitances also increased, leading to the degradation of f_T_ [16,17,18]. To minimize the degradation of f_T_ caused by the use of a high-k material in the passivation layer, we suggest the use of HP and PP structures to improve the frequency characteristics. The AlGaN/GaN HEMT with a properly designed dielectric passivation structure with high V_BD_ and f_T_ is expected to be a good candidate for high-power and high-frequency applications, such as GaN monolithic microwave integrated circuit power amplifiers for military radars and GaN radio frequency (RF) electronic devices for 5^th^ generation mobile telecommunication and autonomous driving.

## 2. Materials and Methods

**Figure 1:**
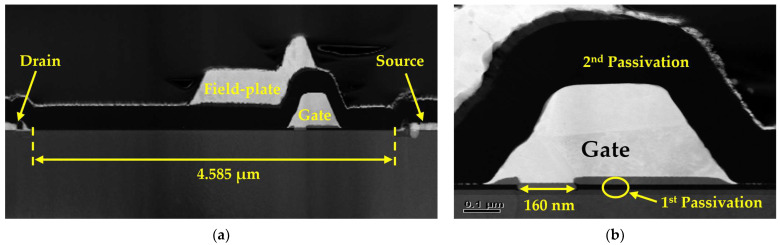
Transmission electron microscope images of the fabricated 0.16 μm gate foot length of the basic Si_3_N_4_ entire passivation (EP) high-electron-mobility transistor (HEMT): (**a**) a cross-sectional view of the unit device and (**b**) an enlarged image of the gate electrode.

A 0.16 μm T-gate AlGaN/GaN HEMT was fabricated, and a cross-sectional view of the unit device is shown in Figure 1a. Figure 1b shows a magnified image of the gate electrode, featuring a 0.16 μm gate foot opening in the 1st passivation layer, which is covered on top by a 2nd passivation layer.

**Figure 2:**
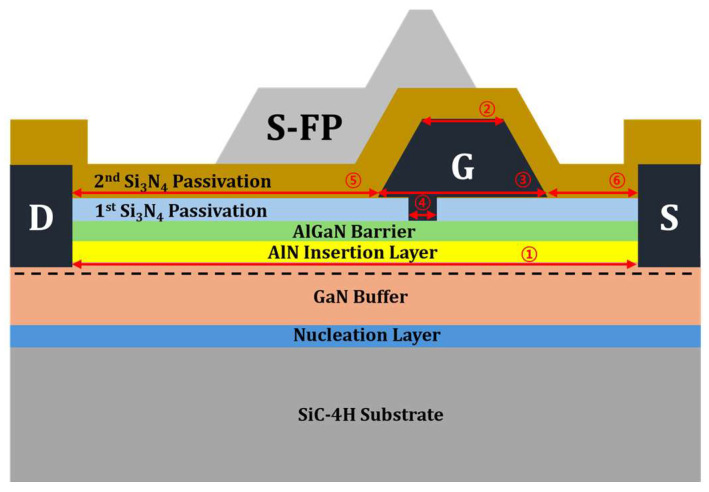
An illustration of 0.16 μm gate foot length of the basic Si_3_N_4_ EP HEMT used for TCAD modeling. S-FP stands for source-connected field plate. G, D, and S stand for gate, drain, and source, respectively.

Figure 2 illustrates a cross-sectional view of a 0.16 μm T-gate AlGaN/GaN HEMT, which was used for technology computer-aided design (TCAD) modeling. In this figure, S is the source electrode, S-FP is the source-connected field plate, G is the gate electrode, and D is the drain electrode. The specific geometric parameters of the modeled device are listed in Table 1.

The AlGaN/GaN HEMT was grown on top of a 4-inch SiC-4H substrate using metal–organic chemical vapor deposition. The epitaxial layers were sequentially stacked and grown as follows: a 20 nm thick nucleation layer, a 1.04 μm thick Fe-doped GaN buffer layer, a 1 nm thick AlN insertion layer, and an 18 nm thick AlGaN barrier layer with 28% Al composition. The Ti/Au/Ni/Au alloyed ohmic contacts for the source and drain electrodes were formed by rapid thermal annealing at 775 °C for 30 s. Device isolation was achieved via P^+^ ion implantation. Subsequently, a 20 nm thick Si_3_N_4_ layer was deposited on the AlGaN barrier layer using plasma-enhanced chemical vapor deposition (PECVD). The first metal interconnection with the source and drain electrodes was established by Ti/Au evaporation after the etching of the 1st Si_3_N_4_ passivation layer. A planar gate was then created using single-layer electron beam lithography. A gate foot opening of 0.16 μm was achieved by exposing a polymethyl methacrylate resist to an electron beam, followed by the removal of the 1st Si_3_N_4_ passivation layer beneath the gate foot opening pattern through dry etching using inductively coupled plasma. The planar gate was defined using a Ni/Au metal stack deposited via electron–beam evaporation and subsequent lift-off processes. After defining the gate shape, a 250 nm thick 2nd Si_3_N_4_ passivation layer was deposited for device passivation using PECVD. The source-connected field plate (S-FP) was formed using a Ti/Au metal lift-off process. Finally, wafer thinning and backside via-hole processes were conducted [19].

To accurately predict the operational characteristics of a device, it is crucial to apply appropriate simulation parameters, such as electrical and thermal parameters, for each epitaxial layer. This meticulous approach ensures reliable and consistent simulation data. Consequently, the simulation parameters were meticulously calibrated to closely align with the actual device operating characteristics. For example, to mitigate the electron punch-through effect and reduce the substrate leakage current, iron (Fe) acceptor trap doping was leveraged in the GaN buffer layer to enhance the V_BD_ [20]. In this simulation, a Gaussian acceptor doping profile was employed, with an acceptor doping concentration of 8.813 × 10^14^/cm^3^ at the AlGaN/GaN interface region and a peak trap concentration of 10^18^/cm^3^ [21]. In addition, a Selberherr impact ionization model was applied to simulate the V_BD_. Other simulation parameters such as electron mobility and heat models were accurately controlled to obtain reliable simulation results. The specific simulation parameters applied to the GaN and AlGaN layers are summarized in Table 2 [22].

After determining the appropriate simulation parameters, simulations were conducted to analyze the direct current (DC) and RF characteristics. The transconductance equation can be expressed as follows:

(1) $$g_{m}=\frac{{\partial I}_{DS}}{\partial V_{GS}},$$

where $g_{m}$, $I_{DS}$, and $V_{GS}$ denote the transconductance, drain current, and gate voltage, respectively. The electric displacement was explained by Equation (2), as follows:

(2) D=εE,

where D, ε, and E denote the electrical displacement, dielectric constant of the material, and electric field, respectively. Before evaluating the frequency characteristics of each structure, the relationship between the parasitic capacitances, such as C_GS_ and C_GD_, and the frequency characteristics was investigated as follows:

(3) $$C=\frac{\varepsilon A}{d},$$

where A and d denote the overlapped area between two electrodes and the distance between the electrodes, respectively.

Next, f_T_ can be determined using Equation (4), as follows:

(4) $$f_{T}=\frac{g_{m}}{2\pi (C_{GS}+C_{GD})}\approx \frac{g_{m}}{2\pi C_{GS}},$$

where $C_{GS}$ and $C_{GD}$ denote the gate-to-source capacitance and gate-to-drain capacitance, respectively. As described in Equation (4), $C_{GS}$ and $C_{GD}$ have an inverse relationship with f_T_, which makes it crucial to minimize parasitic capacitances to maximize the frequency characteristics [23]. Therefore, we propose various dielectric passivation structures using materials with different dielectric constants, such as Si_3_N_4_, Al_2_O_3_, and HfO_2_, to analyze the RF characteristics related to capacitances. The specific material characteristics of these materials are summarized in Table 3 below [24,25].

## 3. Results

### 3.1. Matching Simulated and Measured Data for the Basic Si_3_N_4_ Entire Passivation Structure

**Figure 3:**
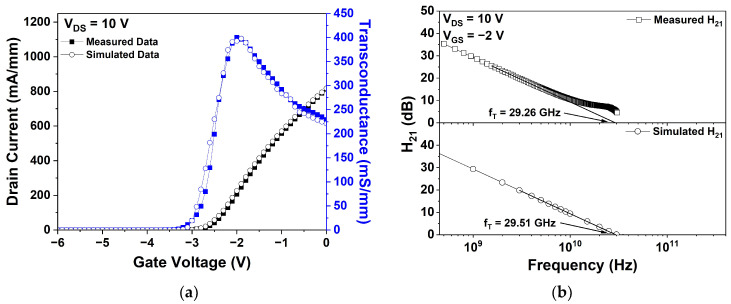
Measured and simulated results of the basic Si_3_N_4_ EP HEMT: (**a**) drain current–gate voltage (I_DS_-V_GS_) transfer characteristics at drain voltage (V_DS_) = 10 V; (**b**) cut-off frequency (f_T_) at V_DS_ = 10 V and gate voltage (V_GS_) = −2 V.

To validate the simulation accuracy, a comparative analysis was conducted between the simulated and measured drain current–gate voltage (I_DS_-V_GS_) transfer characteristics of the fabricated Si_3_N_4_ EP structure device. The measured and simulated data exhibited close agreement in terms of I_DS_ at V_GS_ = 0 V (I_dss_), maximum transconductance (G_m_), and threshold voltage (V_th_). Figure 3a compares the measured and simulated I_DS_-V_GS_ transfer characteristics. The measured and simulated I_dss_ values were 817.10 and 811.99 mA/mm, respectively. Similarly, the measured and simulated maximum transconductance values were 400.39 and 397.65 mS/mm, respectively. Furthermore, the measured V_th_ was −3.1 V, and the simulated value was −3 V. These results confirm a close match between the measured and simulated data for I_dss_, G_m_, and V_th_ with error rates of 0.6%, 0.7%, and 3.2%, respectively.

The measured and simulated f_T_ values of the basic Si_3_N_4_ EP structure are shown in Figure 3b. The RF characteristics were evaluated under V_DS_ = 10 V and V_GS_ = −2 V conditions for both measurement and simulation. More specifically, f_T_ was defined as the intersection of the extension line at the current gain point (H_21_) with the *x*-axis with a slope of −20 dB/decade [26]. The measured and simulated f_T_ values were 29.26 and 29.51 GHz, respectively, demonstrating excellent agreement with the minimal error rate of 0.9%.

### 3.2. Comparative Analysis of Entire Passivation Structures Based on Dielectric Materials

**Figure 4:**
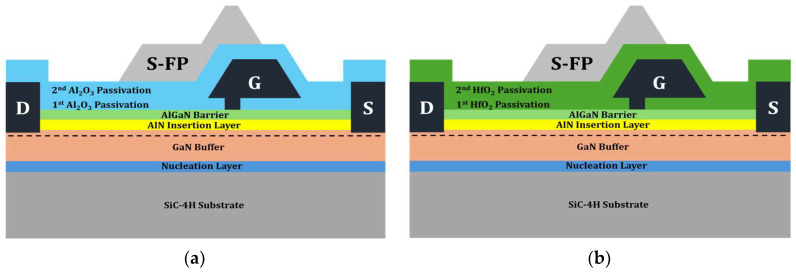
Illustrations of EP structures: (**a**) with Al_2_O_3_; (**b**) with HfO_2_. S-FP stands for source-connected field plate. G, D, and S stand for gate, drain, and source, respectively.

To accommodate high-power applications, the passivation layer of the Si_3_N_4_ EP structure was replaced with a high-k material. Two distinct dielectric materials were modeled (Al_2_O_3_ and HfO_2_) for the EP structure, as shown in Figure 4. All structural parameters except for the passivation material remained unchanged during the simulation.

#### 3.2.1. Simulation Results of the DC Characteristics

**Figure 5:**
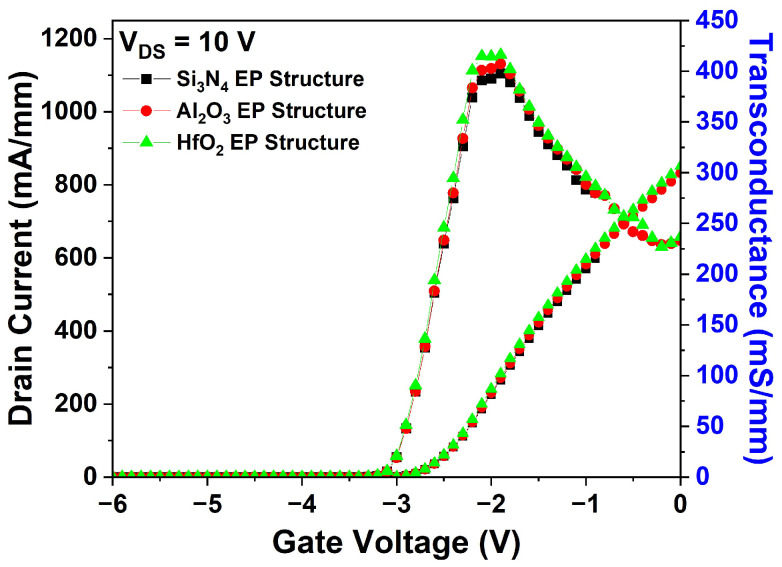
Simulation results of I_DS_-V_GS_ transfer characteristics for the three EP structures at V_DS_ = 10 V.

The DC characteristics of the Al_2_O_3_ and HfO_2_ EP structures were compared with those of the basic Si_3_N_4_ EP structure. Figure 5 shows the I_DS_-V_GS_ transfer characteristics of the three structures at V_DS_ = 10 V. No significant variations in the I_DS_ were observed, and the V_th_ remained constant at −3.0 V.

**Figure 6:**
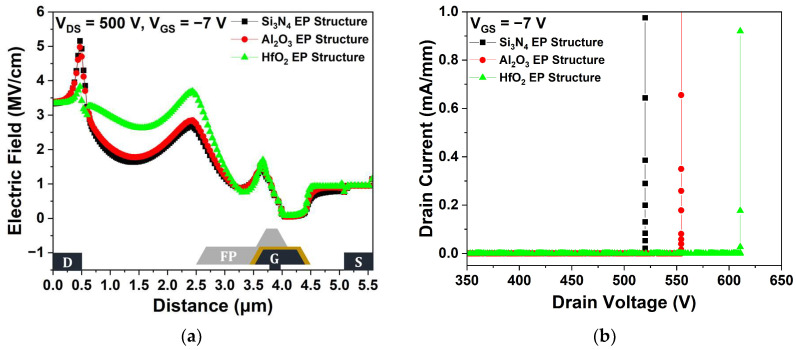
(**a**) Electric field distributions across the 2-DEG (2-dimensional electron gas) channel layer between the source and drain electrodes at V_DS_ = 500 V and V_GS_ = −7 V; (**b**) breakdown voltage (V_BD_) at pinch-off (V_GS_ = −7 V).

Figure 6a shows the electric field distributions within the channel layer at V_DS_ = 500 V for the three passivation structures. Compared with the Si_3_N_4_ EP structure, the Al_2_O_3_ and HfO_2_ EP structures exhibited more efficient electric field dispersion, resulting in a lower maximum electric field in the channel layer due to their high dielectric constant. As the maximum electric field increased, the impact ionization that caused the generation of electron–hole pairs was enhanced; therefore, electric field redistribution effectively improved V_BD_ [27]. The dielectric constant of Al_2_O_3_ is lower than that of HfO_2_, resulting in a relatively lower V_BD_ [28]. The V_BD_ characteristics were simulated under a V_GS_ = −7 V pinch-off condition to ensure a completely off device state. We defined V_BD_ as the V_DS_ when the I_DS_ exceeded 1 mA/mm after completely turning off the device by applying a voltage of −7 V across the gate. As shown in Figure 6b, the Si_3_N_4_, Al_2_O_3_, and HfO_2_ EP structures exhibited V_BD_ values of 519.97, 554.39, and 610.70 V, respectively. The V_BD_ of the Al_2_O_3_ and HfO_2_ EP structures were improved by 6.62% and 17.45%, respectively, compared with that of the Si_3_N_4_ EP structure.

#### 3.2.2. Simulation Results of the RF Characteristics

**Figure 7:**
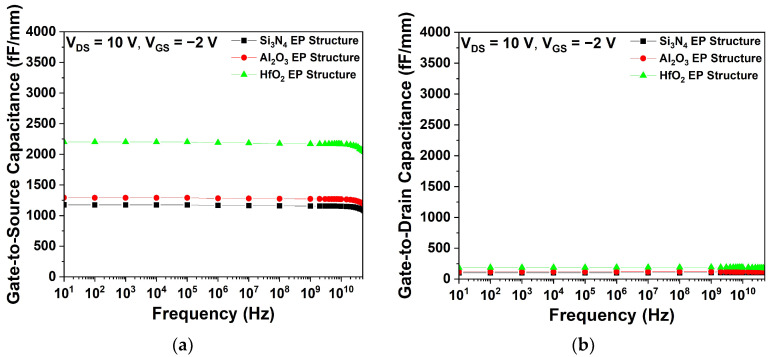
Simulated capacitance characteristics as a function of frequency for three different EP structures at V_DS_ = 10 V and V_GS_ = −2 V; (**a**) gate-to-source capacitance (C_GS_) and (**b**) gate-to-drain capacitance (C_GD_).

Figure 7 shows the parasitic capacitance characteristics of the Si_3_N_4_, Al_2_O_3_, and HfO_2_ EP structures. As shown in Figure 7a,b, the C_GS_ and C_GD_ were determined at V_DS_ = 10 V and V_GS_ = −2 V. The HfO_2_ EP structure exhibited the highest C_GS_ and C_GD_ values, which can be attributed to the dielectric constant of HfO_2_, as described by Equation (3). Conversely, the Al_2_O_3_ EP structure exhibited lower parasitic capacitance values than the HfO_2_ EP structure, due to its lower dielectric constant.

**Figure 8:**
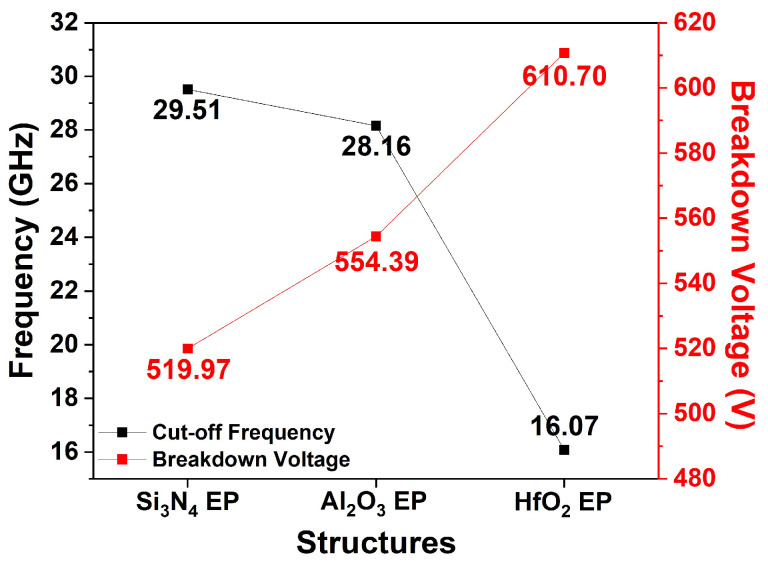
Simulated f_T_ and V_BD_ for three different EP structures.

Figure 8 shows the simulated f_T_ and V_BD_ values for the three EP structures. f_T_ simulations were conducted at V_DS_ = 10 V and V_GS_ = −2 V. According to Equation (4), the f_T_ values of the three EP structures were affected by transconductance (g_m_) and C_GS_. The Si_3_N_4_, Al_2_O_3_, and HfO_2_ EP structures exhibited f_T_ values of 29.51, 28.16, and 16.07 GHz, respectively. Compared with the Si_3_N_4_ EP structure, the Al_2_O_3_ and HfO_2_ EP structures exhibited reductions of 4.57% and 45.54%, respectively.

#### 3.2.3. Simulation Results of the Hybrid Passivation Structure

**Figure 9:**
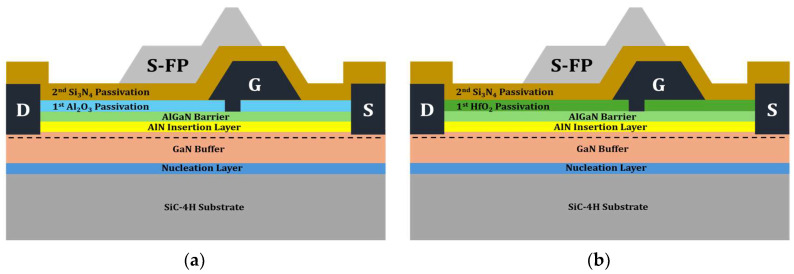
Illustrations of hybrid passivation (HP) structures: (**a**) with Al_2_O_3_; (**b**) with HfO_2_.

To address the trade-off between enhanced V_BD_ values and degraded f_T_ associated with the application of Al_2_O_3_ and HfO_2_ to the EP structures, HP structures were proposed by employing Al_2_O_3_ and HfO_2_ into the 1st passivation and Si_3_N_4_ into the 2nd passivation. Figure 9 shows the schematics of the HP structures with Al_2_O_3_ and HfO_2_.

**Figure 10:**
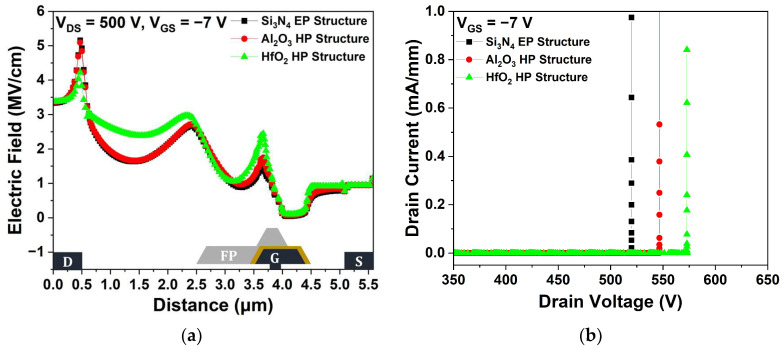
Comparison of Si_3_N_4_ EP, Al_2_O_3_ HP, and HfO_2_ HP structures: (**a**) electric field distributions across the 2-DEG channel layer between the source and drain electrodes at V_DS_ = 500 V and V_GS_ = −7 V; (**b**) V_BD_ at pinch-off (V_GS_ = −7 V).

Figure 10a shows the electric field distributions for three different structures. The maximum electric fields of the Al_2_O_3_ and HfO_2_ HP structures were lower than those of the basic Si_3_N_4_ EP structure. The dielectric constant of Al_2_O_3_ is lower than that of HfO_2_, resulting in a relatively lower V_BD_. Specifically, the V_BD_ values of the Al_2_O_3_ and HfO_2_ HP structures were 546.39 and 572.87 V, respectively, as shown in Figure 10b. However, compared with the EP structure, the HP structure exhibited a reduced V_BD_ because of the use of a high-k material only for the 1st passivation.

**Figure 11:**
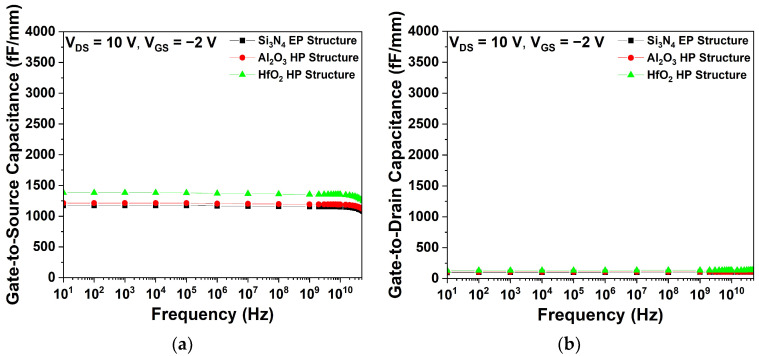
Simulated capacitance characteristics as a function of frequency for Si_3_N_4_ EP, Al_2_O_3_ HP, and HfO_2_ HP structures at V_DS_ = 10 V and V_GS_ = −2 V; (**a**) C_GS_ and (**b**) C_GD_.

Figure 11 shows the parasitic capacitance characteristics of the different 1st passivation materials. Given that L_Gate-Source_ was shorter than L_Gate-Drain_, C_GS_ exhibited a larger value than C_GD_, indicating that the capacitance was affected by the distance between the electrodes [29]. Figure 11 shows that the HfO_2_ HP structure exhibited the highest C_GS_ and C_GD_ values. Conversely, the Al_2_O_3_ HP structure exhibited lower parasitic capacitance values than the HfO_2_ HP structure, which was due to the relatively low dielectric constant of Al_2_O_3_.

**Figure 12:**
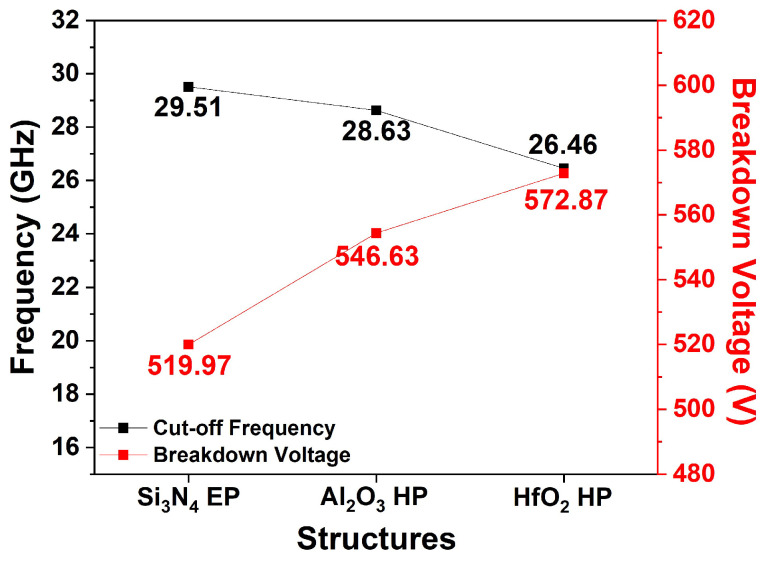
Simulated f_T_ and V_BD_ values for the Si_3_N_4_ EP, Al_2_O_3_ HP, and HfO_2_ HP structures.

Figure 12 compares the simulated f_T_ values for the three dielectric passivation structures. Simulations conducted at V_DS_ = 10 V and V_GS_ = −2 V revealed f_T_ values of 28.63 and 26.46 GHz for the Al_2_O_3_ and HfO_2_ HP structures, respectively. Compared with the Si_3_N_4_ EP structure, these values represent f_T_ reductions of 2.98% and 10.34% for the HfO_2_ and Al_2_O_3_ HP structures, respectively. According to Equation (4), the decrease in f_T_ can be attributed to the increase in C_GS_. Compared to the high-k EP structure, the HP structure compensated for the decrease in RF characteristics by applying a high-k material only at the 1st passivation layer.

### 3.3. Comparative Analysis of Partial Passivation Structures Based on Al_2_O_3_ and HfO_2_

**Figure 13:**
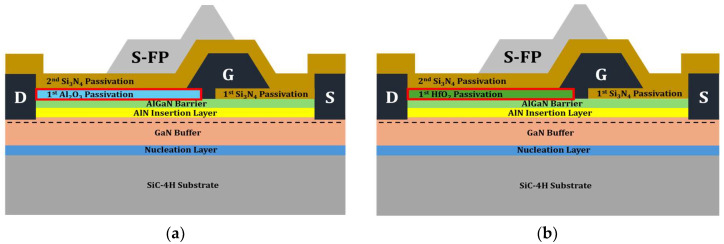
Illustrations of partial passivation (PP) structures: (**a**) with Al_2_O_3_; (**b**) with HfO_2_.

To mitigate the degradation of the RF characteristics observed in the HP structure while preserving the benefits of high-k materials, a PP structure was introduced. By implementing the HP structure, the RF characteristics were improved compared to the high-k EP structure. However, the f_T_ of the HP structure was lower than that of the basic Si_3_N_4_ EP structure. To minimize the degradation of the RF characteristics, we applied the PP structure with a high-k material only for the 1st passivation layer at the drain-gate region. Figure 13 shows schematic diagrams of the PP structure with Al_2_O_3_ and HfO_2_.

#### 3.3.1. Simulation Results of the DC Characteristics

**Figure 14:**
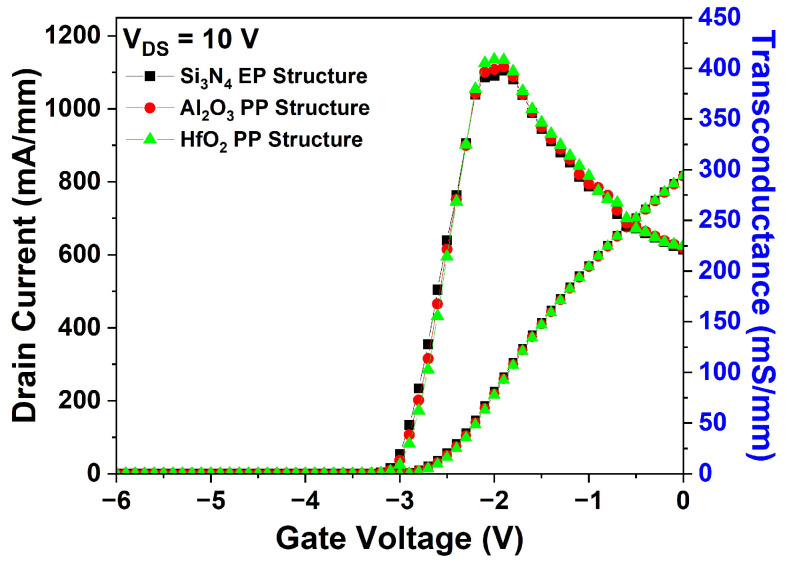
Simulation results of I_DS_-V_GS_ transfer characteristics in the Si_3_N_4_ EP, Al_2_O_3_ PP, and HfO_2_ PP structures.

The I_DS_-V_GS_ transfer characteristics, electric field distribution, and V_BD_ characteristics of the Al_2_O_3_ and HfO_2_ PP structures were simulated. As shown in Figure 14, the I_DS_, g_m_, and V_th_ values remained unaffected by variations in the material of the 1st passivation layer at the drain–gate region.

**Figure 15:**
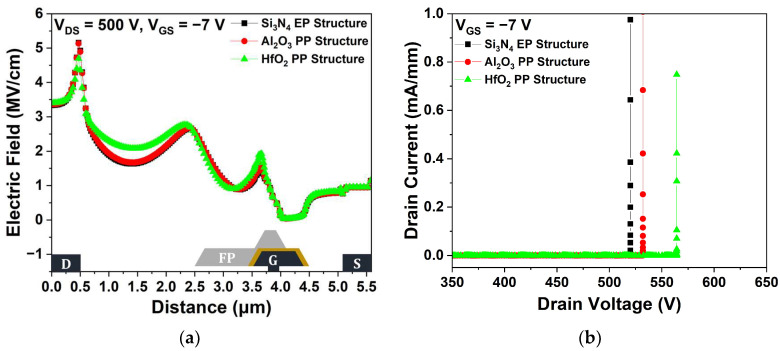
Comparison of Si_3_N_4_ EP, Al_2_O_3_ PP, and HfO_2_ PP structures: (**a**) electric field distributions across the 2-DEG channel layer between the source and drain electrodes at V_DS_ = 500 V and V_GS_ = −7 V; (**b**) V_BD_ at pinch-off (V_GS_ = −7 V).

Figure 15a shows that the maximum electric field for the HfO_2_ PP structure, which exhibited the highest dielectric constant, decreased and was dispersed in the drain–gate region. Conversely, the lower dielectric constant of Al_2_O_3_ in the Al_2_O_3_ PP structure resulted in less pronounced electric field dispersion. Figure 15b shows that the V_BD_ of the HfO_2_ PP structure exhibited the highest V_BD_ value of 564.27 V, while the Si_3_N_4_ EP and Al_2_O_3_ PP structures exhibited comparable values of 519.97 and 532.08 V, respectively. Notably, the use of a high-k material as the 1st passivation layer at the drain–gate region, where the electric field peak occurs, resulted in a slight decrease in V_BD_ for the PP structure compared to the HP structure.

#### 3.3.2. Simulation Results of the RF Characteristics

**Figure 16:**
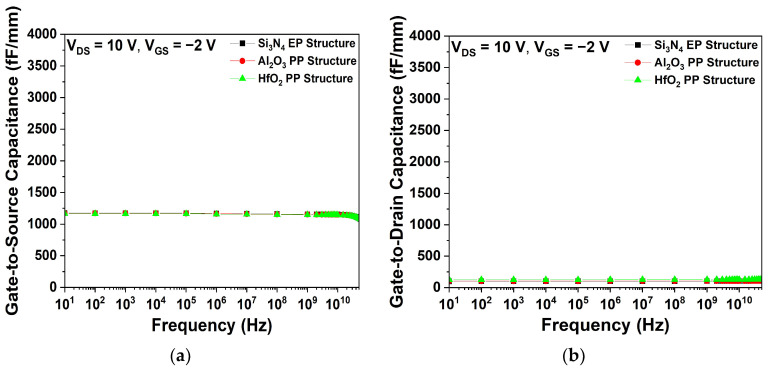
Simulated capacitance characteristics as a function of frequency for Si_3_N_4_ EP, Al_2_O_3_ PP, and HfO_2_ PP structures at V_DS_ = 10 V and V_GS_ = −2 V; (**a**) C_GS_ and (**b**) C_GD_.

Figure 16 shows the parasitic capacitance characteristics of the Si_3_N_4_ EP, HfO_2_, and Al_2_O_3_ PP structures. Given that all three structures employed Si_3_N_4_ as a passivation layer at the source–gate region, the C_GS_ remained consistent, as shown in Figure 16a. However, Figure 16b shows that the HfO_2_ PP structure exhibits the highest C_GD_ value.

**Figure 17:**
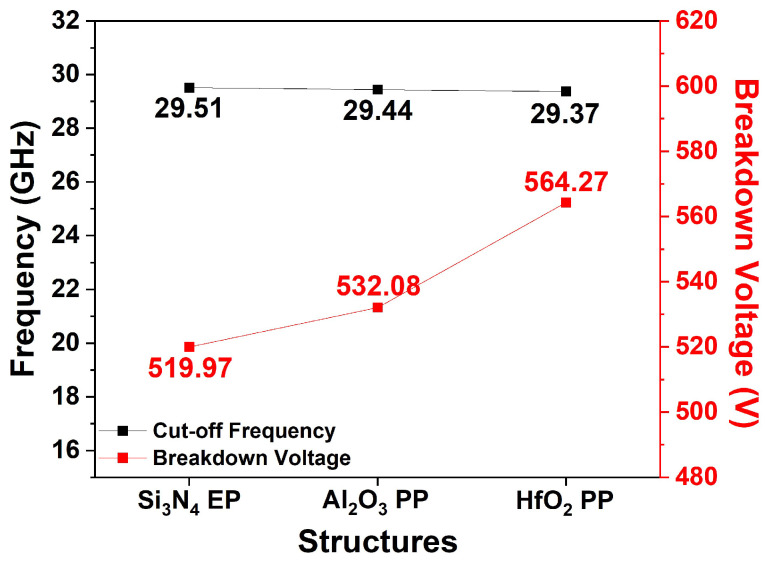
Simulated f_T_ and V_BD_ for Si_3_N_4_ EP, Al_2_O_3_ PP, and HfO_2_ PP structures.

Figure 17 shows the simulated f_T_ values for different dielectric passivation structures at V_DS_ = 10 V and V_GS_ = −2 V. Notably, the f_T_ values of the Si_3_N_4_ EP, Al_2_O_3_ HP, and HfO_2_ HP structures exhibited minimal variations (29.51, 29.44, and 29.37 GHz, respectively). Equation (4) indicates that f_T_ is primarily influenced by C_GS_, and a negligible change in C_GS_ results in the observed f_T_ consistency. These results highlight the effectiveness of the PP structure in mitigating the degradation of the RF characteristics.

## 4. Discussion

This study simulates and analyzes the DC and RF characteristics of various dielectric passivation structures. Table 4 summarizes the DC and RF characteristics, including JFOM, for seven different dielectric passivation structures of the AlGaN/GaN HEMT. Among the Si_3_N_4_, Al_2_O_3_, and HfO_2_ EP structures, the HfO_2_ EP structure exhibited the highest V_BD_. However, the high-k passivation layer inevitably entailed a decrease in f_T_ due to parasitic capacitance. To minimize the degradation of f_T_, HP and PP structures were applied. The JFOM was calculated to analyze the trade-off relationship between V_BD_ and f_T_. The basic Si_3_N_4_ EP structure has a JFOM of 15.34 THz-V. The JFOMs with three different Al_2_O_3_ passivation structures were not significantly different from the Si_3_N_4_ EP structure. However, the proposed HfO_2_ PP structure exhibited the highest JFOM of 16.75 THz-V with enhanced V_BD_ while maintaining f_T._

## 5. Conclusions

This study investigates the DC and RF characteristics of AlGaN/GaN HEMTs using various passivation material configurations via TCAD simulation. The simulation parameters were obtained by matching the simulation data with the measured data of a fabricated basic Si_3_N_4_ EP structure of HEMT to ensure the reliability of the simulation results. The JFOM was calculated to assess the operational characteristics of each proposed dielectric passivation structure considering the trade-off between the breakdown voltage and cut-off frequency. Consequently, based on the highest calculated JFOM among the investigated structures, the HfO_2_ PP structure was proposed as the optimal dielectric passivation structure for achieving superior breakdown voltage and frequency characteristics. This structure shows promise for high-power and high-frequency AlGaN/GaN HEMT applications.

## Figures and Tables

**Table 1:** Specific geometric parameters of the 0.16 μm gate foot length of the basic Si_3_N_4_ EP HEMT.

Parameters	Value (μm)
① L_Source-Drain_	4.585
② L_Gate-Head-Top_	0.26
③ L_Gate-Head-Bottom_	0.71
④ L_Gate-Foot_	0.16
⑤ L_Gate-Drain_	3.175
⑥ L_Gate-Source_	0.7
SiC-4H substrate	5
Nucleation layer	0.02
GaN buffer	1.04
AlN insertion layer	0.001
AlGaN barrier	0.018
1st passivation	0.02
2nd passivation	0.25

**Table 2:** Material parameters for the simulation at a room temperature.

Parameters	Units	GaN	AlGaN
Bandgap energy	eV	3.39	3.88
Electron affinity	eV	4.2	2.3
Dielectric constant	-	9.5	9.38
Low-field electron mobility	-	Farahmand modified Caughey–Thomas Model	
High-field electron mobility	-	GANSAT Model	
Electron saturation velocity	cm/s	1.9 × 10^7^	1.12 × 10^7^
Hole saturation velocity	cm/s	1.9 × 10^7^	1.00 × 10^6^
Electron Shockley–Read–Hall lifetime	s	1.0 × 10^−8^	1.0 × 10^−8^
Hole Shockley–Read–Hall lifetime	s	1.0 × 10^−8^	1.0 × 10^−8^

**Table 3:** Material characteristics of Si_3_N_4_, Al_2_O_3_, and HfO_2_.

Parameters	Units	Si_3_N_4_	Al_2_O_3_	HfO_2_
Dielectric constant	-	~7.5	~9	~25
Bandgap energy	eV	5.3	7.1	5.8

**Table 4:** A summary of DC and RF characteristics of various dielectric passivation structures of HEMT.

Parameters	Units	Si_3_N_4_	Al_2_O_3_			HfO_2_		
Structure type	-	EP	EP	HP	PP	EP	HP	PP
Peak electric field	MV/cm	5.16	4.98	5.09	5.13	3.83	4.22	4.69
Breakdown voltage (V_BD_)	V	519.97	554.39	546.63	532.08	610.70	572.87	564.27
Cut-off frequency (f_T_)	GHz	29.51	28.16	28.63	29.44	16.07	26.46	29.37
Johnson’s figure-of-merit (JFOM)	THz-V	15.34	15.63	15.65	15.66	9.81	15.16	16.75

## Data Availability

Data are contained within the article.
